# Comparison of Clinical and Pathological Characteristics Between Extremely Multiple GGNs and Single GGNs

**DOI:** 10.3389/fonc.2021.725475

**Published:** 2021-09-21

**Authors:** Xin Wang, Manqi Wu, Haifeng Shen, Yuntao Nie, Kai Zhang, Zihan Wei, Ziyang Wang, Fan Yang, Kezhong Chen

**Affiliations:** Department of Thoracic Surgery, Peking University People’s Hospital, Peking University, Beijing, China

**Keywords:** ground-glass nodule, clinical characteristics, prognosis, lung cancer, multiple

## Abstract

**Objective:**

This study aims to compare the clinical and pathological characteristics between patients undergoing surgery for extremely multiple ground-glass nodules (GGNs) and those for single GGN.

**Methods:**

We defined extremely multiple GGNs as follows: (i) number of GGNs ≥3, (ii) GGN diameter between 3 and 30 mm, and (iii) no less than three nodules that were surgically removed and pathologically diagnosed. Patients with extremely multiple GGNs and single GGNs who underwent surgery at the same time were retrospectively analyzed. The patients were divided into three groups according to the number of nodules: exceedingly multiple nodules (EMN) group (>10), highly multiple nodules (HMN) group (three to 10), and single nodule (SN) group. The clinical and pathological characteristics, surgical methods and prognosis were analyzed.

**Results:**

Ninety-nine patients with single nodules and 102 patients with extremely multiple nodules were enrolled. Among the patients with extremely multiple nodules, 43 (42.2%) had >10 nodules. There were no significant differences in demographic characteristics, such as age, sex, and smoking history, between the groups, but there were differences in tumor characteristics. All patients with >10 nodules showed bilateral pulmonary nodules and presented with both pure and mixed GGNs. The single GGNs were smaller in diameter, and the proportion of mixed GGNs and pathologically invasive adenocarcinoma was lower than that of the primary nodules in the exceedingly multiple GGNs group (*p* < 0.05). However, the proportion of both mixed GGNs and malignant nodules decreased significantly with the increasing number of total lesions. During postoperative follow-up, one patient in the highly multiple nodules group had a local recurrence, and 16 (15.7%) patients in the extremely multiple GGNs group and 10 (9.8%) patients in the single GGN group had enlarged unresected GGNs or additional GGNs.

**Conclusions:**

Our study revealed the clinical and pathologic characteristics, surgical methods, and prognosis of patients with extremely multiple GGNs and compared them with those of patients with a single GGN. Although the primary nodules in extremely multiple GGNs may have higher malignancy than those in the single nodule group, the proportion of both mGGNs and malignant nodules decreased significantly with the increasing number of lesions, and the prognosis of patients with extremely multiple GGNs was satisfied.

## Introduction

Ground-glass nodules (GGNs) are nonspecific radiologic findings showing hazy opacity, without blocking the underlying pulmonary vessels or bronchial structures, on high-resolution computed tomography (HRCT) (1) and are classified as either pure ground-glass nodules (pGGNs) or mixed ground-glass nodules (mGGNs) according to the absence or presence of solid components. The pathology of GGNs can be malignant or benign and be associated with conditions such as inflammation, focal interstitial fibrosis, and hemorrhage (2).

The detection rate of GGN has steadily increased in recent years due to the widespread use of low-dose computed tomography, ranging from 2.7 to 95.5% in some lung cancer screening trials (3–7). In addition, GGNs frequently appear as extremely multiple nodules (≥3), which has made the diagnosis and treatment of extremely multiple GGNs a research hot spot (8, 9). Previous research has confirmed that GGNs are more likely to be detected in young female non-smokers (8) and more likely to be malignant (10). However, no scholars have reported the characteristics of extremely multiple nodules and compared these nodules with single GGNs. Furthermore, there exists some controversy about how to treat patients with GGNs.

We collected the clinical and pathology data of 201 patients who were diagnosed with extremely multiple GGNs (≥3) or single GGNs who underwent surgery in recent years for retrospective analysis. The objective of this study was to understand the characteristics, surgical method, and prognosis of patients undergoing surgery for extremely multiple GGNs.

## Methods

### Patients

**Figure 1:**
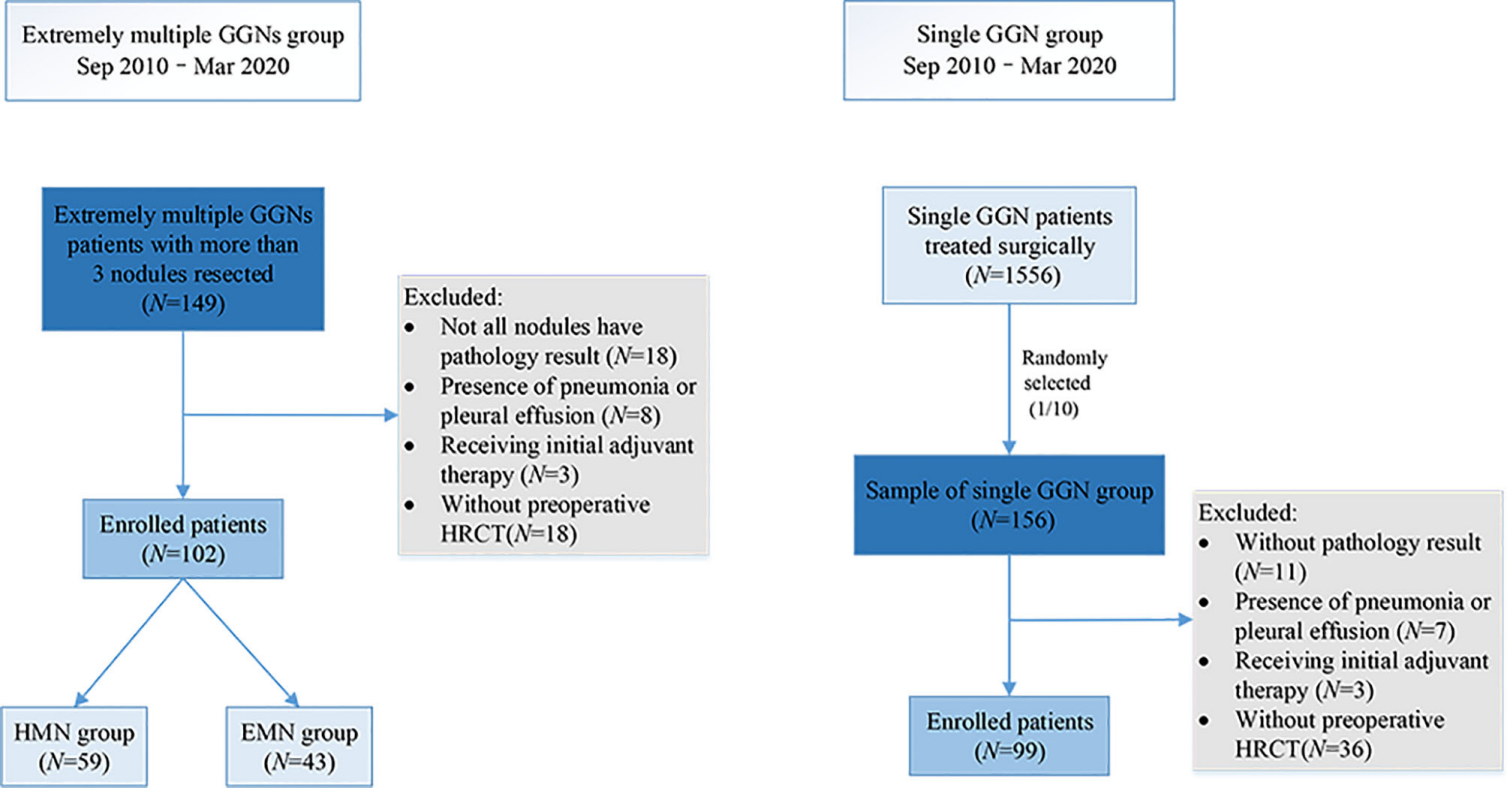
Enrollment flow chart of the study.

From September 2010 to March 2020, 1,556 patients with single GGNs and 149 patients with extremely multiple GGNs underwent surgery in our center. A total of 156 of the 1,556 (one out of 10) patients with single GGNs were randomly selected as the sample to match the number of patients with extremely multiple nodules in a ratio of 1:1. The data of the 156 patients with single GGNs and all the patients with extremely multiple GGNs was collected. The inclusion criteria for the single nodule (SN) group were as follows: (i) only one GGN was found on preoperative imaging and (ii) the GGN diameter was between 3 and 30 mm. The inclusion criteria for the extremely multiple nodules group were as follows: (i) number of GGNs ≥3, (ii) GGN diameter between 3 and 30 mm, and (iii) no less than three nodules were surgically removed and pathologically diagnosed. The patients in the extremely multiple nodules group were divided into two subgroups according to the number of nodules: the exceedingly multiple nodules (EMN) group (>10) and the highly multiple nodules (HMN) group (three to 10) (**Figure 1**). The primary nodule in the group with extremely multiple GGNs was defined as the main tumor to be surgically resected as decided upon by the surgeons, which primarily depended upon its radiologic invasiveness, the percentage of solid components, and the tumor size. All patients were regularly evaluated by HRCT scans from the day of surgery; the intervals were at the discretion of the attending physician. Generally, the patients were reviewed every 3 months within 2 years after surgery, every 6 months starting in the third year, and annually starting in the fifth year. The components of the follow-up review included chest CT, abdominal ultrasound and tumor markers, and brain magnetic resonance imaging. Whole-body bone scan imaging was considered according to the condition of the patient.

This study was reviewed and approved by the Institutional Review Board of Peking University People’s Hospital.

### Radiologic Evaluation

All CT scans were performed on 16-detector CT scanners and obtained at 120 kVp and 40–60 mA, with rotation times of up to 1 s. Images were reconstructed at 1.0-mm slice width with the use of the standard mediastinal (width, 350 HU; level, 40 HU) and lung (width, 1,500 HU; level, 600 HU) window width and window level settings. The diameter of the tumor was defined as the largest axial diameter of the nodule in the lung window setting. All image data were extracted independently by two thoracic radiologists.

### Surgical Approach

With reference to corresponding guidelines and literature, different treatment strategies (follow-up or resection) were performed according to the characteristics of the pulmonary nodules (11–13). Our basic surgical strategies for multiple pulmonary nodules were as follows: (1) preliminary assessment of the location and the size of the nodules, imaging features, and estimation of postoperative respiratory function; (2) when the nodules were all pure GGNs, sublobar resection (wedge and segmental resection) was preferred, while in cases of subsolid nodules, lobectomy of the main solid nodules and sublobar resection of other scattered nodules were performed as per the usual practice. All palpable ipsilateral nodules were resected simultaneously. Unless GGNs were deeply embedded, they could not be resected by wedge resection; and (3) for contralateral tumors, two-stage surgery was generally recommended. If all contralateral lesions were small pure GGNs, a wait-and-see approach was adopted, and growth and invasion were monitored. Similarly, for single GGNs, sublobar resection was preferred when a sufficient margin could be assured, especially for peripheral ground glass opacity-dominant nodules smaller than 2 cm (14, 15).

### Histologic Evaluation

All clinical specimens were examined and recorded by two pathology specialists. Lung adenocarcinoma was analyzed according to the World Health Organization classification (16), and each nodule was reviewed for size, location, pleural invasion, and lymphatic invasion according to the International Association for the Study of Lung Cancer (IASLC) (17).

### Statistical Analysis

For most variables, we calculated descriptive statistics, such as medians with interquartile ranges (for data with skewed distribution) and proportions (percentages). Statistical comparisons between groups were evaluated by *t*-test, analysis of variance, Mann–Whitney *U*-tests, and Kruskal–Wallis tests when appropriate. To explore the variation of the variable with the increasing number of nodules, the Loess method was used for smoothing curve fitting. Data for the interval between surgical resection and the last follow-up visit were analyzed *via* the Kaplan–Meier method using confirmed recurrence deaths to calculate the recurrence-free survival and the overall survival. *P*-values less than 0.05 were considered to be statistically significant. The statistical analyses were performed using R, version 3.5.3.

## Results

### Clinical Characteristics

Ninety-nine patients with single nodules and 102 patients with extremely multiple nodules were enrolled in the study (**Figure 1**), and the median number of nodules in the group with extremely multiple nodules was 10 (range, 3–82). Fifty-nine (55.6%) patients were in the HMN group, and 43 (44.4%) were in the EMN group. The characteristics and a comparison among the three groups are summarized in **Table 1**. There was a greater proportion of female patients in the HMN and EMN groups than in the SN group, although the difference between the two groups was not yet significant (*p* = 0.06). The mean age of the HMN group patients was 55.2 ± 8.9 years, while that of the EMN group patients was 57.1 ± 9.8 years (*p* = 0.29). Most patients had no complaints, no positive tumor markers, and no family history or smoking history, and these characteristics did not differ significantly among the three groups.

**Table 1:** Clinical characteristics of the patients.

Variable	Extremely multiple ground-glass nodules (GGNs)				SN group (*N* = 99)	*P* ^b^
	HMN group (*N* = 59)	EMN group (*N* = 43)	*P* ^a^	Overall (*N* = 102)		
Sex (%)			0.64			0.06
Female	44 (74.6)	34 (79.1)		78 (76.5)	63 (63.6)	
Complaint (%)			0.47			0.73
Yes	11 (18.6)	11 (25.6)		22 (21.6)	19 (19.2)	
Age			0.29			0.83
Mean (SD)	55.2 (8.9)	57.1 (9.8)		56.0 (9.1)	55.7 (9.5)	
Family history (%)			0.72			0.35
Yes	4 (6.8)	4 (9.3)		8 (7.8)	12 (12.1)	
Smoking history (%)			1.00			0.31
Yes	10 (16.9)	7 (16.3)		17 (16.7)	11 (11.2)	
Emphysema (%)			0.70			0.79
Yes	5 (8.5)	2 (4.7)		7 (6.9)	8 (8.1)	
Positive tumor markers (%)			0.03			0.61
Yes	9 (15.3)	15 (34.9)		24 (23.5)	20 (20.2)	
Nodule distribution (%)			0.02			NA
Bilateral	51 (86.4)	43 (100.0)		66 (91.7)	NA	
Nodule type (%)			<0.01			NA
pGGN only	13 (22.0)	0 (0.0)		13 (12.7)	43 (43.4)	
mGGN only	4 (6.8)	0 (0.0)		4 (3.9)	56 (56.6)	
Both pGGN and mGGN	42 (71.2)	43 (100.0)		85 (83.3)	NA	
Bilateral surgery (%)			0.06			NA
Yes	26 (44.1)	11 (25.6)		23 (34.8)	NA	
Operation method (%)			0.77			NA
Sublobar resection	36 (61.0)	24 (55.8)		60 (58.8)	77 (77.8)	
Lobectomy	8 (13.6)	5 (11.6)		13 (12.7)	22 (22.2)	
Both	15 (25.4)	14 (32.6)		29 (28.4)	NA	
Lymph node (%)			0.27			0.87
No intervention	4 (6.8)	3 (7.0)		7 (6.9)	9 (9.1)	
Dissection	21 (35.6)	22 (51.2)		43 (42.2)	40 (40.4)	
Sampling	34 (57.6)	18 (41.9)		52 (51.0)	50 (50.5)	
Operation interval			0.27			NA
Median (IQR)	5.0 (4.0, 7.0)	4.0 (4.0, 4.5)		4.0 (4.0, 6.0)	NA	
Resection rate			<0.01			NA
Median (IQR)	75.0% (57.1%, 100.0%)	33.3% (25.8%, 46.3%)		50.0% (33.3%, 77.7%)	NA	

HMN, highly multiple nodules; EMN, exceedingly multiple nodules; SN, single nodule; SD, standard deviation; IQR, interquartile range.

a Comparison between the HMN group and the EMN group.

b Comparison between patients with extremely multiple GGNs and those with a single GGN.

NA, not available.

**Figure 2:**
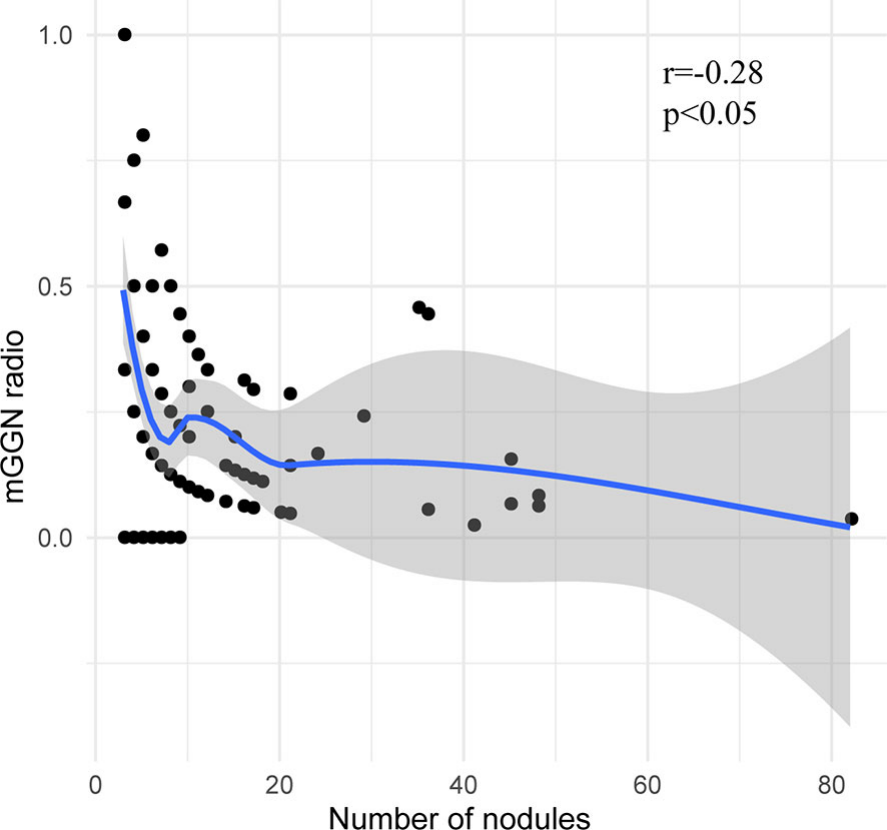
The correlation between the proportion of mixed ground-glass nodules and the total number of nodules.

**Figure 3:**
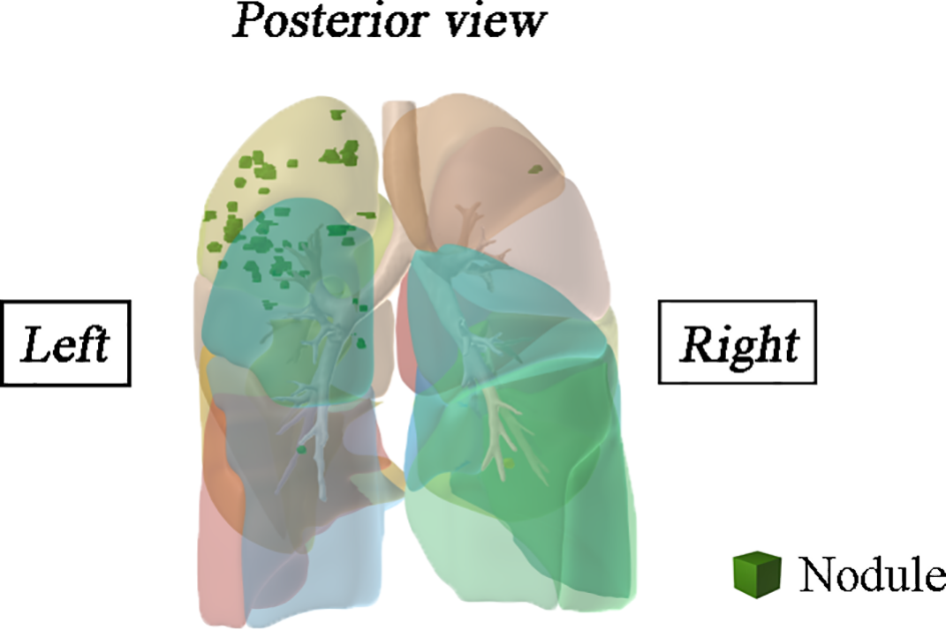
Spatial location information of the multifocal samples of a patient was reconstructed in three-dimensional.

A total of 1,344 GGNs were detected in the HMN and EMN groups, including 1,078 pure GGNs and 266 mixed GGNs. Most of the nodules were located in the upper lobe of the right lung and the upper lobe of the left lung (32.7 and 29.1%, respectively), among which 68.6% were in the apical and posterior segments (**Table 2**). The median diameters of the primary nodules in the SN group, HMN group, and EMN group were 11.0 mm (range, 4.0–29.0 mm), 12.8 mm (range, 6.6–30.0 mm), and 17.2 mm (range, 10.2–30.0 mm), respectively. The proportions of mGGNs in the primary nodules were 56.6, 62.7, and 83.7% for the SN, HMN, and EMN groups, respectively. There were significant differences in the size and the type of the primary nodules between the SN group and the EMN group (*p* < 0.01), while the differences in the other pairwise comparisons (the SN group *vs*. the HMN group and the HMN group *vs*. the EMN group) were not significant after Bonferroni correction (Bonferroni-adjusted significance threshold, *p* <0.017) (**Table 3**). However, the proportion of mGGNs decreased significantly as the number of total lesions increased (correlation coefficient, *r* = -0.28, **Figure 2**). In the EMN group, all patients presented with bilateral nodules, with both pGGNs and mGGNs showing a wider distribution (100 *vs*. 85%, *p* = 0.03) and higher imaging heterogeneity (100 *vs*. 75%, *p* = 0.004) than the HMN group. Interestingly, in four patients, the nodules were clustered, and no less than 10 GGNs were concentrated in a single segment (**Figure 3**). The other characteristics did not differ significantly between the two groups.

**Table 2:** Percentage distribution of the pulmonary nodules.

		Right superior lobe	Right middle lobe	Right inferior lobe	Left superior lobe	Left inferior lobe	*P*
Total nodules	EMN	31.96%	8.68%	16.67%	30.44%	12.26%	0.147
	HMN	32.94%	7.45%	18.04%	26.67%	14.90%	
	SN	39.39%	4.04%	23.23%	19.19%	14.14%	
Malignant nodules	EMN	29.27%	9.76%	30.49%	13.41%	17.07%	0.233
	HMN	29.81%	8.65%	18.27%	26.92%	16.35%	
	SN	37.80%	4.88%	21.95%	23.17%	12.20%	

**Table 3:** Analysis of the characteristics of primary nodules.

	Single nodule group (*n* = 99) *n* (%)	Highly multiple nodules group (*n* = 59) *n* (%)	Exceedingly multiple nodules group (*n* = 43) *n* (%)	*P*	*P* ^a^
Distribution				0.45	NA
Superior lobes	58 (58.6)	37 (62.7)	30 (69.8)		
Type				<0.01	<0.01
mGGN	56 (56.6)^a^	37 (62.7)	36 (83.7)^a^		
Size				<0.01	<0.01
Median (IQR)	11.0 (8.0, 17.5)^a^	12.8 (12.8, 18.5)	17.2 (17.2, 23.3)^a^		
Operation method				0.01	0.01
Sublobar resection	77 (77.8)^a^	36 (61.0)	24 (55.8)^a^		
Pathological pattern				0.01	0.01
IA	44 (44.4)^a^	36 (61.0)	30 (69.8)^a^		

IA, invasive adenocarcinoma.

a The two groups were considered significantly different, with a P-value below 0.017 after Bonferroni correction for multiple pairwise comparisons (α = 0.05, n = 3), whereas in the other pairwise comparisons, there was no significant difference.

### Surgical Approach

All patients had the primary nodule excised, while only eight patients with three to five GGNs had all the lesions excised in the extremely multiple nodules group. The nodule resection rate in the EMN group was significantly lower than that in the HMN group (median, 33.3 *vs*. 75.0%, *p* < 0.01). More sublobar resection for primary nodules was performed in the SN group than in the HMN (*p* = 0.02) and EMN (*p* < 0.01) groups, and the difference between the SN group and the EMN group was significant after Bonferroni correction (**Table 3**). As shown in **Table 1**, in the extremely multiple GGNs group, 60 (58.8%) patients underwent sublobar resection (segmentectomy and wedge resection) only, 13 (12.7%) patients underwent lobectomy only, and 29 (28.4%) patients underwent lobectomy combined with sublobar resection. There was no significant difference in the choice of surgical method between the HMN and EMN groups (*p* = 0.77). In 51 patients who presented with bilateral pulmonary nodules, 26 (44.1%) underwent a two-stage bilateral surgery, with a median interval of 4 months (interquartile range, 4–6 months). Only 25.6% of patients in the EMN group had bilateral surgery, which was less than that in the HMN group (44.1%), although the difference between the two groups was not yet significant (*p* = 0.06).

### Pathological Characteristics

**Figure 4:**
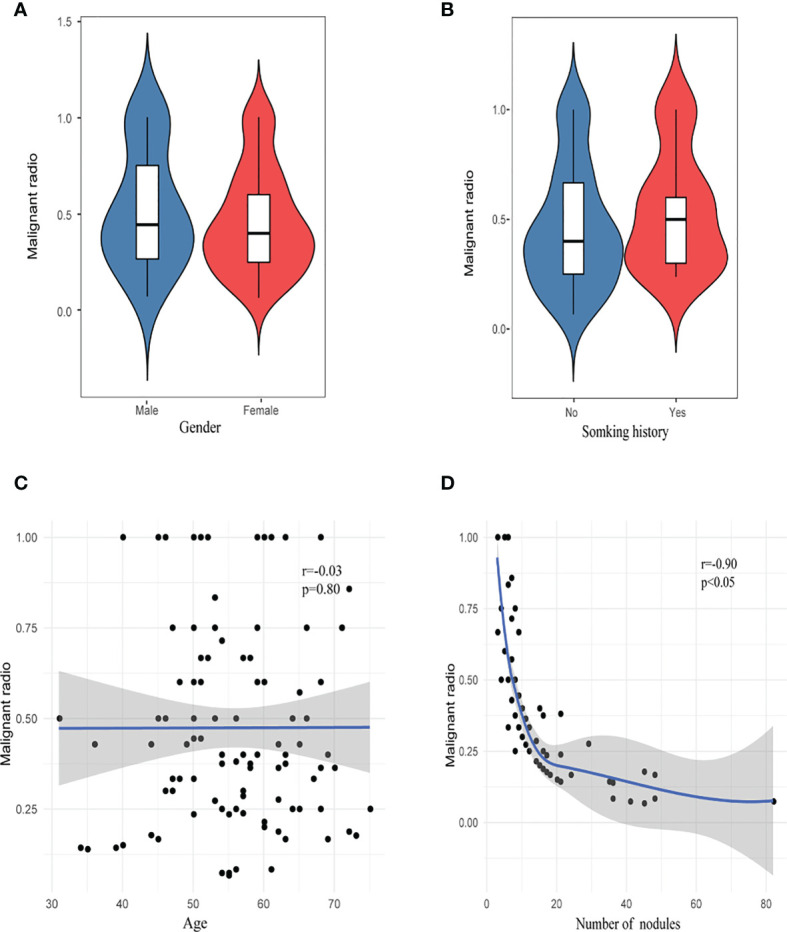
Risk factor analysis of malignant rate. **(A)** Gender, **(B)** smoking history, **(C)** age, and **(D)** number of nodules.

The proportion of invasive adenocarcinoma in the primary nodules of the SN group (44.4%) was less than those in the HMN (61.0%, *p *= 0.04) and EMN groups (69.8%, *p* < 0.01), and the difference between the SN group and the EMN group was significant after Bonferroni correction (**Table 3**). Among the resected nodules of the EMN and HMN groups, the malignant proportion was 73.7%, and the proportions of atypical adenomatoid hyperplasia, adenocarcinoma *in situ* (AIS), micro-invasive adenocarcinoma (MIA), and invasive adenocarcinoma were 7.0, 13.2, 27.8, and 33.7%, respectively. There was no significant difference in the incidence of invasive adenocarcinoma between the two groups (**Table 4**). The proportion of malignant nodules had no significant relationship with the age, sex, or smoking history of the patients but decreased with the increase in the number of nodules (**Figure 4**). Systematic lymph node dissection or lymph node sampling was performed in 94.4% of the patients, and no lymph node invasion was observed, neither in the patients with pure ground-glass nodules nor in those with mixed ground-glass nodules.

**Table 4:** Pathological analysis of ground-glass nodules.

	Highly multiple nodules group (*n* = 220) *n* (%)	Exceedingly multiple nodules group (*n* = 278) *n* (%)	*P*
Benign (except AAH)	44 (20.0)	47 (16.9)	0.375^a^
AAH/AIS/MIA	108 (49.1)	132 (47.5)	0.391^b^
IA	68 (30.9)	99 (35.6)	

AAH, atypical adenomatous hyperplasia; AIS, adenocarcinoma in situ; MIA, minimally invasive adenocarcinoma.

a Comparison between benign lesions and malignant lesions.

b Comparison between IA and AAH/AIS/MIA.

### Prognosis

A total of 4/201 patients were lost at the end of the follow-up period. The median follow-up period was 37 months (range, 13–120 months). No serious postoperative complications or perioperative deaths were observed in any group. Ten patients in the SN group had additional GGNs during postoperative follow-up, and none of them underwent intervention. Nine patients in the HMN group and seven patients in the EMN group had enlarged unresected GGNs or additional GGNs during postoperative follow-up, nine of whom had a second operation on the contralateral side, and the others had no intervention. There was no significant difference in the rate of nodule progression between the HMN group and the EMN group (15.3 *vs*. 16.3%, *p* = 0.88). Three nodules from the three patients who underwent the second surgery were pathologically confirmed to be invasive adenocarcinoma, with no lymph node invasion. One patient in the HMN group who presented preoperatively with a mixed ground-glass nodule with a solid component ratio greater than 50% was found to have a local recurrence 34 months after wedge resection. The pathology of the recurrent nodules was papillary adenocarcinoma. None of the other 200 patients experienced a recurrence and no death was recorded either during the follow-up period.

## Discussion

In this study, 76.5% of the patients in the HMN and EMN groups were female, a slightly higher percentage than that in the SN group (63.6%) and other studies (63.8–71.8%) on single nodules (18, 19). Most patients had no chief complaint, no family history of lung cancer, and no positive tumor markers, which did not differ significantly among the SN group, the EMN group, and the HMN group and was consistent with studies on single GGNs (18, 19). Since our study participants were from the surgical population, a higher proportion of patients had mixed ground-glass nodules. In our study, the proportion of both mGGNs and malignant nodules decreased significantly with the increasing number of nodules, which indicated that patients with extremely multiple GGNs may not have higher malignancy or stronger invasiveness due to the increasing number of GGNs. However, among the primary nodules, compared with the SN group, the proportion of both mGGNs and invasive adenocarcinoma was higher in the HMN and EMN groups, especially in the EMN group. Furthermore, the size of the primary nodules in the EMN group was larger than that in the SN group. Therefore, the primary nodules in the extremely multiple nodules group may have higher malignancy than those in the single nodule group, and patients with extremely multiple GGNs should be assessed mainly for the primary nodules rather than the number of nodules.

Multiple GGNs are considered multiple primary lung cancers and at the early stage of tumorigenesis rather than metastatic tumors, according to the statements from the Fleischner Society and IASLC Staging and Prognostic Factors Committee (20). However, in some patients, most nodules are concentrated in a single segment. Previous studies have shown that four patients had two lesions that shared the same rare mutation (21, 22) identified by whole-exome sequencing. The exact metastatic routes remain unexplored, but metastasis possibly occurred *via* the airway. Therefore, in patients with clustered GGNs, attention should be given to the possibility of metastasis.

The scope of surgery for patients with multiple ground-glass nodules has always been controversial, and it is generally considered that sublobar resection is more appropriate than lobectomy for smaller GGNs and pGGNs. Miller and colleagues compared the outcomes of lobectomy and sublobar resection for tumors ≤10 mm, and they found that there was no significant difference in survival rate or local recurrence rate (23). Lee and colleagues suggested that the surgical approach of pGGN with pathological types of AIS and MIA is recommended to be sublobar resection rather than lobectomy (24). There are also reports in the literature that, for multiple GGN patients with surgical resection, the prognosis is satisfactory; even sublobectomy does not affect the prognosis (25, 26). Compared with the abovementioned studies that only focused on one or two GGNs, our study indicates that sublobar resection for pure GGNs and nonmain lesions also does not affect the prognosis for patients with extremely multiple GGNs, while the study of Nakao found that marginal or primary recurrence occurred in four of 26 GGN patients 5 years after local resection (27). Notably, in our study, a patient with mixed ground-glass nodules relapsed 34 months after sublobar resection. The main lesion in this patient had a few high-risk characteristics, such as a larger size (2.3 cm), worse pathological subtype (papillary adenocarcinoma), and a higher proportion of solid components (>50%). The other four patients with papillary adenocarcinoma underwent lobectomy. In summary, for pure GGNs and nonmain lesions in extremely multiple GGNs, sublobar resection may be a priority, while the clinical imaging characteristics and rapid freezing pathology during the operation are important factors in determining the surgical approach of the main lesions, especially for larger-sized and subsolid nodules.

Regarding whether to remove all nodules at the same time, according to IASLC guidelines, the nodules resected in patients with multiple ground-glass nodules should be considered as having a high probability of malignancy (20), which was confirmed by our study, showing an overall malignant rate of resected nodules at 73.7% and an even higher malignant rate of 89% for mixed ground-glass nodules, consistent with those studies of single GGNs (28). For multiple GGNs, some studies suggest that the main lesion be removed first and that the prognosis will not be affected whether other lesions are removed (25, 29). However, for extremely multiple GGNs, it remains uncertain whether excision of the main lesion alone is sufficient. In our study, the majority of patients (84.3%) did not experience an enlargement of the unresected nodules during postoperative follow-up, consistent with studies on multiple GGNs (84%) (30). The patients with nodule enlargement were treated with continued observation or secondary surgery, while their survival was not affected. Therefore, the survival rate of patients with extremely multiple GGNs may not be affected when the number of resected nodules is appropriately reduced to improve the quality of life. For the remaining GGN lesions, regular follow-up can be recommended; once the solid component of the lesion increases or the volume increases, repeat surgery can be considered.

Previous studies showed that the incidence of lymph node metastasis in GGNs with solid components greater than 50% was 10–26% (31–36). However, Suzuki *et al.* analyzed the data of 545 patients and proposed that, for adenocarcinoma with a diameter ≤3 cm and solid component ratio ≤50%, the specificity for the diagnosis of pathologically non-invasive adenocarcinoma reached 98.7% (37). Hattori *et al.* also reported that systemic lymph node dissection in patients with GGNs did not improve the survival rate (38). In our study, for extremely multiple GGNs, none of the patients had a lymph node invasion, which indicates that the risk of lymph node invasion does not increase with the increasing number of GGNs. Therefore, systematic lymph node dissection may not be necessary for extremely multiple GGNs, especially if with a solid component of less than 50%.

Our study has some limitations and shortcomings. First, the surgical intervention indications and intervention methods of multiple GGNs are still controversial and inconsistent, and the treatment strategy of our center may be different from that of other centers in the world. Second, the median follow-up time was short, and longer follow-ups are needed to further determine whether multiple unresected GGNs will progress in the future.

To the best of our knowledge, this study is the first to reveal the clinical and pathologic features, surgical methods, and prognosis of patients with extremely multiple GGNs and compare them with those of patients with single GGNs. The result shows that, although the primary nodules in extremely multiple GGNs may have higher malignancy than those in the single nodule group, which should be of concern to clinicians, the proportion of both mGGNs and malignant nodules decreased significantly with the increasing number of lesions, and the prognosis of patients with extremely multiple GGNs was satisfactory. Systematic lymph node dissection may not be necessary for extremely multiple GGNs, which will provide insights for the treatment strategy of such patients.

## Data Availability Statement

The original contributions presented in the study are included in the article/supplementary material. Further inquiries can be directed to the corresponding author.

## Ethics Statement

The studies involving human participants were reviewed and approved by Institutional Review Board of Peking University People’s Hospital. The patients/participants provided their written informed consent to participate in this study.

## Author Contributions

XW, MW, HS, YN, ZYW, ZHW, and KZ participated in data collection. XW, MW, ZYW, KC, and FY participated in data analysis. XW, MW, ZYW, YN, and KC wrote the manuscript. XW, MW, KC, and FY revised the manuscript. All authors contributed to the article and approved the submitted version.

## Funding

This study was supported by the National Natural Science Funds (grant number 82072566) and the Peking University People’s Hospital Research and Development Funds (grant number RS2019-01).

## Conflict of Interest

The authors declare that the research was conducted in the absence of any commercial or financial relationships that could be construed as a potential conflict of interest.

## Publisher’s Note

All claims expressed in this article are solely those of the authors and do not necessarily represent those of their affiliated organizations, or those of the publisher, the editors and the reviewers. Any product that may be evaluated in this article, or claim that may be made by its manufacturer, is not guaranteed or endorsed by the publisher.

## References

[1] Hansell DM Bankier AA MacMahon H McLoud TC Müller NL Remy J. Fleischner Society: Glossary of Terms for Thoracic Imaging. Radiology (2008) 246(3):697–722. doi: 10.1148/radiol.2462070712 18195376

[2] Miura A Akagi S Nakamura K Ohta-Ogo K Hashimoto K Nagase S. Different Sizes of Centrilobular Ground-Glass Opacities in Chest High-Resolution Computed Tomography of Patients With Pulmonary Veno-Occlusive Disease and Patients With Pulmonary Capillary Hemangiomatosis. Cardiovasc Pathol (2013) 22(4):287–93. doi: 10.1016/j.carpath.2012.12.002 23312620

[3] Scholten ET de Jong PA de Hoop B van Klaveren R van Amelsvoort-van de Vorst S Oudkerk M. Towards a Close Computed Tomography Monitoring Approach for Screen Detected Subsolid Pulmonary Nodules? Eur Respir J (2015) 45(3):765–73. doi: 10.1183/09031936.00005914 25431271

[4] Henschke CI Yankelevitz DF Mirtcheva R McGuinness G McCauley D Miettinen OS. CT Screening for Lung Cancer: Frequency and Significance of Part-Solid and Nonsolid Nodules. AJR Am J Roentgenol (2002) 178(5):1053–7. doi: 10.2214/ajr.178.5.1781053 11959700

[5] Henschke CI. Early Lung Cancer Action Project: Overall Design and Findings From Baseline Screening. Cancer (2000) 89(11 Suppl):2474–82. doi: 10.1002/1097-0142(20001201)89:11+<2474::AID-CNCR26>3.0.CO;2-2 11147630

[6] Chong S Lee KS Chung MJ Kim TS Kim H Kwon OJ. Lung Cancer Screening With Low-Dose Helical CT in Korea: Experiences at the Samsung Medical Center. J Korean Med Sci (2005) 20(3):402–8. doi: 10.3346/jkms.2005.20.3.402 PMC2782194 15953860

[7] Zhang Y Jheon S Li H Zhang H Xie Y Qian B. Results of Low-Dose Computed Tomography as a Regular Health Examination Among Chinese Hospital Employees. J Thorac Cardiovasc Surg (2020) 160(3):824–31.e4. doi: 10.1016/j.jtcvs.2019.10.145 31987625

[8] Zhang Y Fu F Chen H. Management of Ground-Glass Opacities in the Lung Cancer Spectrum. Ann Thorac Surg (2020) 110(6):1796–804. doi: 10.1016/j.athoracsur.2020.04.094 32525031

[9] Sihoe ADL Petersen RH Cardillo G. Multiple Pulmonary Ground Glass Opacities: Is it Time for New Guidelines? J Thorac Dis (2018) 10(11):5970–3. doi: 10.21037/jtd.2018.10.67 PMC6297419 30622765

[10] Wang J Ma H Ni CJ He JK Ma HT Ge JF. Clinical Characteristics and Prognosis of Ground-Glass Opacity Nodules in Young Patients. J Thorac Dis (2019) 11(2):557–63. doi: 10.21037/jtd.2019.01.32 PMC6409253 30963000

[11] MacMahon H Naidich DP Goo JM Lee KS Leung ANC Mayo JR. Guidelines for Management of Incidental Pulmonary Nodules Detected on CT Images: From the Fleischner Society 2017. Radiology (2017) 284(1):228–43. doi: 10.1148/radiol.2017161659 28240562

[12] Suzuki K. Whack-A-Mole Strategy for Multifocal Ground Glass Opacities of the Lung. J Thorac Dis (2017) 9(Suppl 3):S201–S7. doi: 10.21037/jtd.2017.04.03 PMC5392542 28446985

[13] Niu R Shao X Shao X Wang J Jiang Z Wang Y. Lung Adenocarcinoma Manifesting as Ground-Glass Opacity Nodules 3 Cm or Smaller: Evaluation With Combined High-Resolution CT and PET/CT Modality. AJR Am J Roentgenol (2019) 213(5):W236–w45. doi: 10.2214/AJR.19.21382 31361533

[14] Suzuki K Watanabe SI Wakabayashi M Saji H Aokage K Moriya Y. A Single-Arm Study of Sublobar Resection for Ground-Glass Opacity Dominant Peripheral Lung Cancer. J Thorac Cardiovasc Surg (2020). doi: 10.1016/j.jtcvs.2020.09.146 33487427

[15] Handa Y Tsutani Y Okada M. Transition of Treatment for Ground Glass Opacity-Dominant Non-Small Cell Lung Cancer. Front Oncol (2021) 11:655651. doi: 10.3389/fonc.2021.655651 33937064 PMC8082027

[16] WHO. Classification of Tumours Editorial Board. Thoracic Tumours. Lyon (France: International Agency for Research on Cancer (2021).

[17] Detterbeck FC Franklin WA Nicholson AG Girard N Arenberg DA Travis WD. The IASLC Lung Cancer Staging Project: Background Data and Proposed Criteria to Distinguish Separate Primary Lung Cancers From Metastatic Foci in Patients With Two Lung Tumors in the Forthcoming Eighth Edition of the TNM Classification for Lung Cancer. J Thorac Oncol (2016) 11(5):651–65. doi: 10.1016/j.jtho.2016.01.025 26944304

[18] Ye T Deng L Wang S Xiang J Zhang Y Hu H. Lung Adenocarcinomas Manifesting as Radiological Part-Solid Nodules Define a Special Clinical Subtype. J Thorac Oncol (2019) 14(4):617–27. doi: 10.1016/j.jtho.2018.12.030 30659988

[19] Fu F Zhang Y Wen Z Zheng D Gao Z Han H. Distinct Prognostic Factors in Patients With Stage I Non-Small Cell Lung Cancer With Radiologic Part-Solid or Solid Lesions. J Thorac Oncol (2019) 14(12):2133–42. doi: 10.1016/j.jtho.2019.08.002 31437531

[20] Naidich DP Bankier AA MacMahon H Schaefer-Prokop CM Pistolesi M Goo JM. Recommendations for the Management of Subsolid Pulmonary Nodules Detected at CT: A Statement From the Fleischner Society. Radiology (2013) 266(1):304–17. doi: 10.1148/radiol.12120628 23070270

[21] Li R Li X Xue R Yang F Wang S Li Y. Early Metastasis Detected in Patients With Multifocal Pulmonary Ground-Glass Opacities (GGOs). Thorax (2018) 73(3):290–2. doi: 10.1136/thoraxjnl-2017-210169 PMC5870446 29056599

[22] Li Y Li X Li H Zhao Y Liu Z Sun K. Genomic Characterisation of Pulmonary Subsolid Nodules: Mutational Landscape and Radiological Features. Eur Respir J (2020) 55(2):1901409. doi: 10.1183/13993003.01409-2019 31699841

[23] Miller DL Rowland CM Deschamps C Allen MS Trastek VF Pairolero PC. Surgical Treatment of non-Small Cell Lung Cancer 1 Cm or Less in Diameter. Ann Thorac Surg (2002) 73(5):1545–50; discussion 50-1. doi: 10.1016/S0003-4975(02)03525-7 12022547

[24] Lee HY Lee KS. Ground-Glass Opacity Nodules: Histopathology, Imaging Evaluation, and Clinical Implications. J Thorac Imaging (2011) 26(2):106–18. doi: 10.1097/RTI.0b013e3181fbaa64 21508733

[25] Shimada Y Saji H Otani K Maehara S Maeda J Yoshida K. Survival of a Surgical Series of Lung Cancer Patients With Synchronous Multiple Ground-Glass Opacities, and the Management of Their Residual Lesions. Lung Cancer (Amsterdam Netherlands) (2015) 88(2):174–80. doi: 10.1016/j.lungcan.2015.02.016 25758554

[26] Nakata M Sawada S Yamashita M Saeki H Kurita A Takashima S. Surgical Treatments for Multiple Primary Adenocarcinoma of the Lung. Ann Thorac Surg (2004) 78(4):1194–9. doi: 10.1016/j.athoracsur.2004.03.102 15464469

[27] Nakao M Yoshida J Goto K Ishii G Kawase A Aokage K. Long-Term Outcomes of 50 Cases of Limited-Resection Trial for Pulmonary Ground-Glass Opacity Nodules. J Thorac Oncol (2012) 7(10):1563–6. doi: 10.1097/JTO.0b013e3182641b5c 22878750

[28] Qu R Hao Z Zhang Y Bie L Fu X Zhang N. Single-Center Experience of Simultaneous Bilateral Uni-Portal Video-Assisted Thoracoscopic Surgery for Multiple Ground-Glass Opacities. J Cardiothoracic Surg (2020) 15(1):69. doi: 10.1186/s13019-020-01107-0 PMC7178615 32326944

[29] Gao RW Berry MF Kunder CA Khuong AA Wakelee H Neal JW. Survival and Risk Factors for Progression After Resection of the Dominant Tumor in Multifocal, Lepidic-Type Pulmonary Adenocarcinoma. J Thorac Cardiovasc Surg (2017) 154(6):2092–9.e2. doi: 10.1016/j.jtcvs.2017.07.034 28863952

[30] Chen K Chen W Cai J Yang F Lou F Wang X. Favorable Prognosis and High Discrepancy of Genetic Features in Surgical Patients With Multiple Primary Lung Cancers. J Thorac Cardiovasc Surg (2018) 155(1):371–9.e1. doi: 10.1016/j.jtcvs.2017.08.141 29092754

[31] Asamura H Suzuki K Watanabe S Matsuno Y Maeshima A Tsuchiya R. A Clinicopathological Study of Resected Subcentimeter Lung Cancers: A Favorable Prognosis for Ground Glass Opacity Lesions. Ann Thorac Surg (2003) 76(4):1016–22. doi: 10.1016/S0003-4975(03)00835-X 14529977

[32] Ikeda N Maeda J Yashima K Tsuboi M Kato H Akada S. A Clinicopathological Study of Resected Adenocarcinoma 2 Cm or Less in Diameter. Ann Thorac Surg (2004) 78(3):1011–6. doi: 10.1016/j.athoracsur.2004.03.048 15337040

[33] Suzuki K Kusumoto M Watanabe S Tsuchiya R Asamura H. Radiologic Classification of Small Adenocarcinoma of the Lung: Radiologic-Pathologic Correlation and its Prognostic Impact. Ann Thorac Surg (2006) 81(2):413–9. doi: 10.1016/j.athoracsur.2005.07.058 16427823

[34] Aoki T Tomoda Y Watanabe H Nakata H Kasai T Hashimoto H. Peripheral Lung Adenocarcinoma: Correlation of Thin-Section CT Findings With Histologic Prognostic Factors and Survival. Radiology (2001) 220(3):803–9. doi: 10.1148/radiol.2203001701 11526285

[35] Matsuguma H Yokoi K Anraku M Kondo T Kamiyama Y Mori K. Proportion of Ground-Glass Opacity on High-Resolution Computed Tomography in Clinical T1 N0 M0 Adenocarcinoma of the Lung: A Predictor of Lymph Node Metastasis. J Thorac Cardiovasc Surg (2002) 124(2):278–84. doi: 10.1067/mtc.2002.122298 12167787

[36] Nakata M Sawada S Yamashita M Saeki H Kurita A Takashima S. Objective Radiologic Analysis of Ground-Glass Opacity Aimed at Curative Limited Resection for Small Peripheral non-Small Cell Lung Cancer. J Thorac Cardiovasc Surg (2005) 129(6):1226–31. doi: 10.1016/j.jtcvs.2004.10.032 15942561

[37] Suzuki K Koike T Asakawa T Kusumoto M Asamura H Nagai K. A Prospective Radiological Study of Thin-Section Computed Tomography to Predict Pathological Noninvasiveness in Peripheral Clinical IA Lung Cancer (Japan Clinical Oncology Group 0201). J Thorac Oncol (2011) 6(4):751–6. doi: 10.1097/JTO.0b013e31821038ab 21325976

[38] Hattori A Matsunaga T Takamochi K Oh S Suzuki K. Significance of Lymphadenectomy in Part-Solid Lung Adenocarcinoma: Propensity Score Matched Analysis. Ann Thorac Surg (2018) 106(4):989–97. doi: 10.1016/j.athoracsur.2018.04.069 29852148

